# Electrophysiological model of human temporal contrast sensitivity based on SSVEP

**DOI:** 10.3389/fnins.2023.1180829

**Published:** 2023-07-31

**Authors:** Tsvetomira Tsoneva, Gary Garcia-Molina, Peter Desain

**Affiliations:** ^1^Department of Digital Engagement, Cognition and Behavior, Philips Research, Eindhoven, Netherlands; ^2^Centre for Cognition, Donders Institute for Brain, Cognition and Behaviour, Radboud University, Nijmegen, Netherlands; ^3^Sleep Number Labs, San Jose, CA, United States; ^4^Center for Sleep and Consciousness, University of Wisconsin, Madison, WI, United States

**Keywords:** SSVEP, electrophysiology, psychophysics, perception, temporal, contrast sensitivity, TCSF, flicker

## Abstract

The present study aims to connect the psychophysical research on the human visual perception of flicker with the neurophysiological research on steady-state visual evoked potentials (SSVEPs) in the context of their application needs and current technological developments. In four experiments, we investigated whether a temporal contrast sensitivity model could be established based on the electrophysiological responses to repetitive visual stimulation and, if so, how this model compares to the psychophysical models of flicker visibility. We used data from 62 observers viewing periodic flicker at a range of frequencies and modulation depths sampled around the perceptual visibility thresholds. The resulting temporal contrast sensitivity curve (TCSC) was similar in shape to its psychophysical counterpart, confirming that the human visual system is most sensitive to repetitive visual stimulation at frequencies between 10 and 20 Hz. The electrophysiological TCSC, however, was below the psychophysical TCSC measured in our experiments for lower frequencies (1–50 Hz), crossed it when the frequency was 50 Hz, and stayed above while decreasing at a slower rate for frequencies in the gamma range (40–60 Hz). This finding provides evidence that SSVEPs could be measured even without the conscious perception of flicker, particularly at frequencies above 50 Hz. The cortical and perceptual mechanisms that apply at higher temporal frequencies, however, do not seem to directly translate to lower frequencies. The presence of harmonics, which show better response for many frequencies, suggests non-linear processing in the visual system. These findings are important for the potential applications of SSVEPs in studying, assisting, or augmenting human cognitive and sensorimotor functions.

## 1. Introduction

Repetitive visual stimulation (RVS), in the form of luminance modulation or pattern reversal, at frequencies between 1 and 100 Hz, triggers a specific brain response in the electroencephalogram (EEG), known as steady-state visual evoked potentials (SSVEPs). SSVEPs manifest as oscillatory components at the fundamental and harmonic frequencies of the RVS and are most prominent over the areas close to the visual cortex. Their high signal-to-noise (SNR) ratio and robustness to artifacts make them an important tool that can be used in cognitive and clinical neurosciences and neural engineering. SSVEPs can be a sensitive electrophysiological index of several visual-perceptual and cognitive functions and can provide the means for studying brain rhythmic activity and functional brain connectivity. They are also widely used in overt and covert attention-based brain-computer interfaces (BCIs).

In SSVEP experiments, the characteristics of the response are influenced by the properties of the stimuli, which may play a crucial role in the interpretation of the findings. However, certain perceptual aspects are often overlooked, which can introduce methodological confounds and lead to alternative interpretations of the findings. Furthermore, the use of repetitive visual stimulation presents application-specific challenges concerning subjects safety and comfort. The relationship between physical stimuli and subjective experiences in highly controlled laboratory settings has been explored in the field of psychophysics using carefully constructed stimuli and procedures to understand perceptual and cognitive processes underlying sensory experience. As such, perceptual insights are sometimes difficult to directly apply in neuroscience experiments. Our objective is to connect the psychophysical research on the human visual perception of flicker with the neurophysiological research on SSVEPs by making psychophysical findings more accessible and applicable to a wider range of SSVEP applications. By doing so, we aim to contribute to the improvement of the design and interpretation of studies that utilize RVS.

An important aspect of visual perception is human sensitivity to contrast, which is the relative difference in stimulus luminance. The visual system is more sensitive to contrast than absolute luminance (Perz, 2019), and this holds for both spatial and temporal contrasts. Michelson contrast (see Equation 1), which defines stimulus modulation depth (MD) is often used in visual perception studies. According to the Michelson's definition, a luminance stimulus, modulated at a specific temporal (or spatial) frequency, has a modulation depth ranging from 0 to 1. If the MD is sufficiently small, the luminance variation cannot be perceived, but when the MD is sufficiently large, the luminance variation becomes visible (Perz, 2019).


(1) $$\begin{array}{l} MD=\frac{L_{max}-L_{min}}{L_{max}+L_{min}}, \end{array}$$

where *L*_*min*_ corresponds to the minimum luminance of the stimulus and *L*_*max*_ corresponds to the maximum luminance of the stimulus.

Luminance modulation of sufficient contrast in the temporal domain gives rise to the perception of flicker. The minimum MD necessary for flicker to be detected is called the *visibility threshold*. Contrast sensitivity is defined as the reciprocal of the visibility threshold. The temporal contrast sensitivity function (TCSF) describes contrast sensitivity as a function of temporal frequency. To derive such a psychophysical model, various experimental paradigms, such as detection tasks, staircase procedure or two-alternative forced choice (2AFC) task, are used. In these experiments, MD is systematically varied, and subjective responses from participants are collected to estimate a detection probability at a specific frequency, which is then used to estimate a visibility threshold (see Section 2.6.4. for more details).

**Figure 1:**
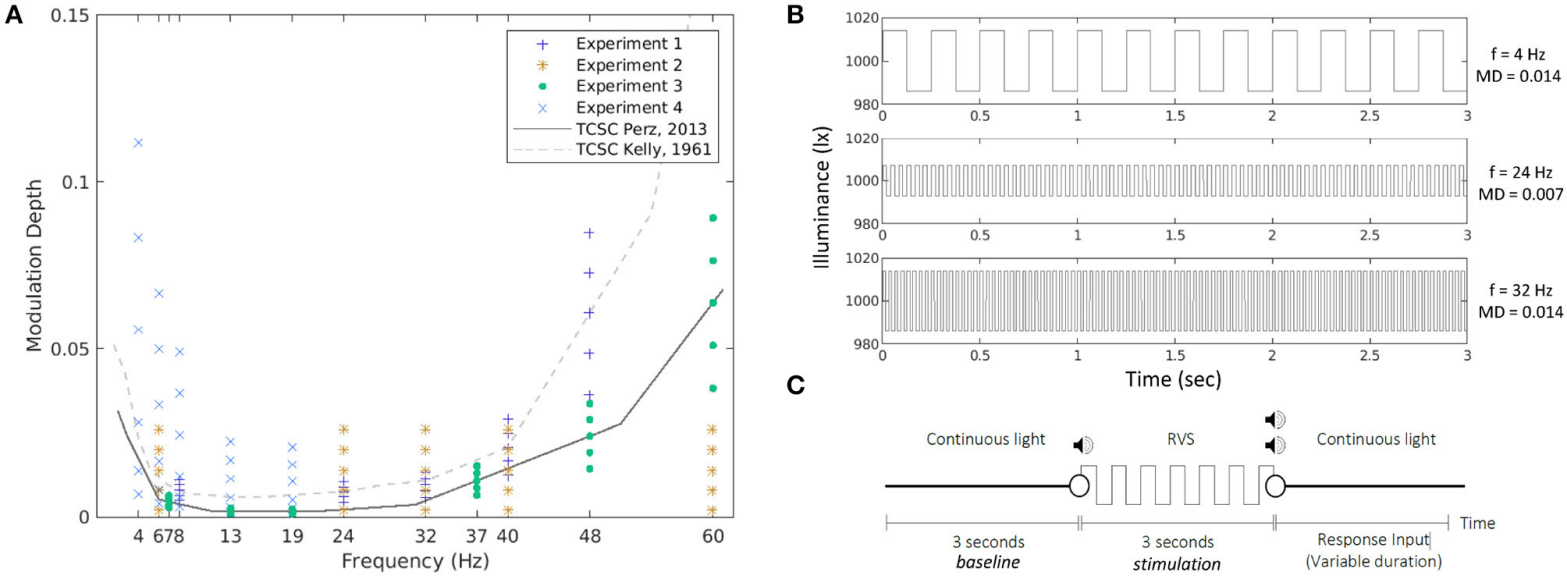
Stimulation conditions in all four experiments together with the two TCSCs used as reference **(A)**. The MD is expressed as a proportion, ranging between 0 and 1. Example waveforms corresponding to three of the conditions tested in the experiments **(B)**. An experimental trial **(C)**.

The TCSF has been measured in psychophysical studies under various conditions (de Lange, 1958; Kelly, 1961; Barten, 1999; Rovamo et al., 1999; Perz et al., 2013; Perz, 2019). The results of the later two publications have been obtained using the same experimental setup as that described in this study. The TCSF depends on luminance, eccentricity, and stimulus size. At higher luminance levels (above 3*cd*/*m*^2^), the curve exhibits band-pass characteristics, peaking in the range between 10 Hz and 20 Hz, and decreasing after that (see the inverse of Kelly, 1961; Perz et al., 2013 in Figure 1A, and Perz, 2019 in Figure 6). Generally, temporal sensitivity increases with increasing luminance in a logarithmic fashion (Watson et al., 1986).

Campbell and Maffei (1970) were among the first to relate the visual evoked potential (VEP) amplitude to psychophysical contrast threshold using 8 Hz counterphase-modulated gratings. Further studies have demonstrated the relationship between contrast sensitivity functions derived from steady-state VEP data and those derived from psychophysics (Harris et al., 1976; Pirchio et al., 1978; Cannon, 1983; Norcia et al., 1985; Hamilton et al., 2021). Although these studies have utilized SSVEPs, their focus has been on *spatial* rather than temporal contrast sensitivity. Usually, a single temporal frequency is employed and spatial contrast is parametrically varied (swept) over a range of values. Some temporal sensitivity properties could be extracted from the results of those studies, but the inherently different methodologies, the various stimulus characteristics, and the limited number of conditions tested would make it impossible to create a consolidated temporal sensitivity model.

Electrophysiological investigations of *temporal* contrast sensitivity have been conducted separately at two levels of the visual system, the retina, using electroretinogram (ERG), and the visual cortex, using visual evoked cortical potentials (VECP) (van der Tweel, 1964; van der Tweel and Verduyn Lunel, 1965; Sokol and Riggs, 1971; Sternheim and Cavonius, 1972; Regan and Beverley, 1973; van der Tweel et al., 1998). For the most part, these studies have related the amplitudes (in microvolts) of the principal component of the response wave to the subjective thresholds reported in psychophysics (Sokol and Riggs, 1971). van der Tweel (1964) reported results from two subjects in the high- and low-frequency ranges at various MDs, showing the occurrence of harmonic components, especially at low frequencies, and discrepancies between electrophysiological and psychophysical data at high frequencies: the retinal and cortical response data were found to decline less sharply than those reported in psychophysics (de Lange, 1958). In another study, van der Tweel and Verduyn Lunel (1965) continued their investigation into the linearity and non-linearity of the visual system, using sinusoidally modulated light as the stimulus and the response of a single occipital lead as the effect. They compared the flicker fusion curve (the highest detectable frequency at a given modulation level) at MD of 25% measured subjectively or electophysiologically in three subjects and found that subjective flicker threshold curves bear unclear relation to the size or shape of the electrophysiological responses. Sokol and Riggs (1971) used square waves as the stimuli and recorded VECPs from two electrodes on the midline (2 and 12 cm above the inion) in three subjects for a series of contrast levels at a range of frequencies in different dark adaptation conditions: 15–53 Hz in photopic conditions (luminance above 3*cd*/*m*^2^) and 5–20 Hz in scotopic conditions (luminance below 0.01*cd*/*m*^2^). Unlike the other studies, they recognized the importance of the results being expressed in terms of the electrical analog of a threshold and chose the percentage of contrast at each frequency necessary to satisfy the constant amplitude value using a linear fit function between the median VECP amplitude and the contrast. They found that the scotopic system has higher contrast thresholds and is restricted to a lower range of frequencies than those in the photopic system. The eye appeared to be capable of a resolution beyond 60 Hz, as estimated by the ERG responses, while the VECP and psychophysical data appeared to show a limit below 55 Hz. The contrast sensitivity curves showed a steeper negative slope for psychophysical data in the range from 20 to 40 Hz than for the ERG or VECP data. Sternheim and Cavonius (1972), using horizontal square wave (0.75 cpd) counter phase flickering gratings, found a resemblance of both the shape and the absolute sensitivity of the high-frequency portion (above 10 Hz) of the human VECP sensitivity model with the observer's psychophysical sensitivity (obtained at 4° visual field), but they also reported higher variability (from two observers) in the lower range of the curve. The ERG response could be detected at 60 Hz, when no flicker was visible. Good correlation between psychophysical sensitivity and the VECP evoked by patterned but not by uniform stimuli have been reported by other authors as well (Regan, 1968; Campbell and Maffei, 1970; Regan and Beverley, 1973).

These early studies have mostly relied on a single-channel recording of responses from a limited number of participants to modulated light or gratings, using only a few modulation frequencies in usually highly restricted visual fields. Although they were instrumental in establishing the foundations of our understanding of the neural processes underlying temporal contrast sensitivity, a significant gap exists between the early literature and the current state of the art in neurophysiology research, with several factors contributing to this disparity. The advances in the state-of-the-art EEG systems have led to better amplification and improved temporal and spatial resolutions, enabling researchers to capture SSVEP responses up to 100 Hz (Herrmann, 2001). With the broadening of the applications of SSVEPs, the current systems have become inherently more complex as they use sophisticated digital displays, LED lighting, and complex visual stimuli. This offers superior control and flexibility over various stimulus parameters, resulting in improved experimental throughput, enabling the inclusion of a larger number of subjects and a wider range of modulation frequencies. Given these advancements, it is crucial to reevaluate the existing evidence and develop a temporal sensitivity model that fits better the current technologies and applications.

This article aimed to connect the psychophysical research on the human visual perception of flicker with the neurophysiological research on SSVEPs. This can lead to a better understanding of the underlining mechanism behind SSVEPs and help improve the comfort and safety of the setups in various SSVEP applications. This study is a continuation of our previous studies (Tsoneva et al., 2013; Berumen and Tsoneva, 2017) on SSVEPs at the boundaries of visual perception. We particularly focused on two main questions:

(1) Can we define an electrophysiological model of temporal contrast sensitivity using SSVEPs?
(2) Is there a difference between electrophysiological and psychophysical contrast sensitivity models?

## 2. Methods

We conducted four experiments. All the experiments were approved by the Philips internal ethics committee on biomedical experiments and conducted in accordance with the Declaration of Helsinki.

### 2.1. Study participants

A total of 62 healthy adults with normal or corrected to normal vision voluntarily participated in the four experiments (Table 1). They were carefully screened for a history of photosensitivity, seizures, migraine, or headaches, and only participants who did not report any of these conditions were enrolled. They were sufficiently informed about the study's objectives and asked to sign an informed consent form before the start of the experiment.

**Table 1:** Study volunteers.

**Experiment**	**Participants**	**Age mean (SD)**
Experiment 1	10 (5 men, 5 women)	24.0(1.7)
Experiment 2	12 (9 men, 3 women)	range 20 − 26 years
Experiment 3	24 (17 men, 7 women)	26.4(6.0)
Experiment 4	16 (8 men, 8 women)	26.8(5.6)

### 2.2. Experimental setup

The RVS was presented via two LED panels (Lumileds, LUXEON Rebel, dimensions: 57.5 x 57.5 cm) mounted 0.8 m from each other on a frame at a height of 2.5 m from the floor, close to a white wall. Each panel contained four rows of white LEDs and was controlled by an Agilent 33522A function generator via a laptop using a TCP/IP interface. Prior to the experiments, the setup was carefully calibrated to establish a reliable correspondence between the control voltage applied to the function generator and the resulting luminous output. The guidelines outlined in Schakel et al. (2021) were followed when performing this calibration process.

The light stimulation was reflected on a white wall with a fixation cross in the middle. It covered a total area of 210 x 360 cm (vertically x horizontally). The participants were seated at a distance of 1 m from the wall and had a visual angle of 137°. They were instructed to look at the fixation cross during the experiment and to attempt to avoid movement and blinking during stimuli presentation periods. During preparation (i.e., EEG setup and task explanation), the lights were set to the average light level of the experiment, ensuring light adaptation of at least 20 min.

### 2.3. Stimuli

The repetitive visual stimuli used in the experiments were square waves at frequencies and modulation depths sampled around the psychophysical temporal contrast sensitivity curve (TCSC). Figure 1A presents all stimulation conditions and the two curves used as reference. In the first experiment, we used the TCSC of Kelly (1961), adapted for square waves (Campbell and Robson, 1968), measured at retinal illuminance of 9,300 td, which is the closest to the illuminance levels of our study. For each frequency (8, 28, 32, 40, and 48 Hz), we selected five MDs as fractions of the sensitivity thresholds defined at that frequency, namely, 0.6, 0.8, 1.0, 1.2, and 1.4 times the sensitivity threshold, respectively. In the second experiment, we opted for absolute MDs at 0.002, 0.008, 0.014, 0.020, and 0.026 for each of the five frequencies (6, 24, 32, 40, and 60 Hz, respectively). In the third experiment, we used as a reference the TCSC of Perz et al. (2013), which controls better for flicker adaptation and was estimated using our experimental setup. We measured the SSVEP response for six frequencies (7, 13, 19, 37, 48, and 60 Hz) at five MDs selected as fractions of the sensitivity thresholds defined at that frequency (0.6, 0.8, 1.0, 1.2, and 1.4 times the sensitivity threshold, respectively). The fourth experiment was similar to experiment 3 but measured at five different frequencies (4, 6, 8, 13, and 19 Hz) at six MDs (0.4, 0.8, 1.6, 3.2, 4.8, and 6.4 times the sensitivity threshold at 4 Hz and 0.8, 1.6, 3.2, 6.4, 9.6, and 12.8 times the sensitivity threshold for the other four frequencies, respectively). Those conditions were chosen to cover the expected response space based on the results from the previous experiments. For an overview see Table 2. The TCSC of Perz et al. (2013) was later adapted in Perz (2019), which is used in the rest of the article.

**Table 2:** Stimulation conditions.

	**Frequencies**	**MDs**
Exp. 1	8, 24, 32, 40, 48 Hz	0.6, 0.8, 1.0, 1.2 and 1.4 x TCSC of Kelly (1961)
Exp. 2	6, 24, 32, 40, 60 Hz	0.002, 0.008, 0.014, 0.020 and 0.026 absolute MD
Exp. 3	7, 13, 19, 37, 48, 60 Hz	0.6, 0.8, 1.0, 1.2 and 1.4 x TCSC of Perz et al. (2013)
Exp. 4	4, 6, 8, 13, 19 Hz	0.6, 0.8, 1.0, 1.2 and 1.4 x TCSC of Perz et al. (2013)

The waveforms had a duration of 3 s (6,248 samples at a sample rate of 2,048 Hz). The average light luminance level was 1,000 lx, and the color temperature was 6,500 K. The waveforms were created as defined in Equation 2


(2) $$\begin{array}{l} waveform=AvgLL+square\left(2\times \pi \times t\times F\right)\times MD\times AvgLL, \end{array}$$

where *AvgLL* is the average luminance (1,000 lx), *t* is time in seconds, *F* is the frequency of stimulation in Hz, and *MD* is the modulation depth as proportion, ranging between 0 and 1. The MATLAB (2019) *square* function was used to create the square waves.

Figure 1B shows a few example waveforms used in the experiments.

### 2.4. Experimental task

All waveforms corresponding to the conditions selected in each experiment were presented 10 times each. This resulted in 250–300 trials per experiment. Each trial started with 3 s of continuous light, followed by 3 s of RVS, and finished with another period of continuous light of a variable duration. Because some stimuli were below the perception threshold, the start of RVS presentation was marked by a beep, and the end by two beeps. After the second beep (except in Experiment 1), the participants were expected to indicate whether they had perceived a flicker by pressing a button on a number pad. See Figure 1C for a visual representation of the experimental trial.

To control for adaptation and fatigue effects, all trials were presented in a randomized order, and depending on the experiment, they were presented in two or three blocks of equal trial number. Each block lasted between 14 and 18 min and was followed by a break of variable duration (3–10 min).

### 2.5. Data acquisition

The 32-channel EEG was recorded using the BioSemi ActiveTwo signal acquisition system (BioSemi B.V., Amsterdam, Netherlands) at a sampling frequency of 2,048 Hz. The system uses Ag/AgCl active electrodes, an added *common mode sense* and *driven right leg* as “ground” electrodes. The electrodes were mounted on an elastic cap according to the 32-channel extension of the international 10–20 positioning system. The signal from a photodiode (directed to the wall) was jointly recorded with the EEG for synchronization purposes.

### 2.6. Data analysis

#### 2.6.1. EEG preprocessing

The recorded signals were analyzed using custom MATLAB (2019) scripts and the open-source EEGLAB toolbox (Delorme and Makeig, 2004). First, a 50 Hz notch filter was applied to attenuate the power line interference. Then, the signals were resampled at 256 Hz to reduce the computation time, and a 2-Hz high-pass FIR filter was applied to reduce the baseline shift. Eye blinks and other ocular artifacts were removed by performing independent component analysis (ICA) (Nunez and Srinivasan, 2006) by excluding the component that showed the highest average spatial difference between the front and back electrodes. Then, the signals were referenced to a common average reference (CAR).

The resulting signals were segmented into pairs of 3-s long epochs: baseline, starting 3 s before the stimulus onset, and stimulation starting at stimulus onset, resulting in two epochs for each trial. The epochs were screened one more time for extremely high-amplitude values, high frequency muscle noise, and other irregular artifacts generated by non-cerebral activity by an automatic artifact rejection procedure, limiting the variance over epochs for each condition to the mean plus two times the standard deviation. The proportion of rejected trials was 3.8% on average with a maximum of one trial per condition.

#### 2.6.2. SSVEP response and threshold estimate

Both baseline and stimulation epochs were analyzed in the frequency domain using the fast Fourier transform (FFT) in windows of one second with no overlap, resulting in three windows per epoch and a frequency resolution of 1 Hz. The log-transformed mean over the resulting three windows was, then, used to calculate the *z*-*score* and *Cohen*′*sd* score.

*Cohen*′*sd* (see Equation 3) is a measure of the standardized mean difference between two distributions, and we used it to find the harmonic component, which differentiates the best between baseline and stimulation epochs, for each stimulation frequency (see Equation 4).


(3) $$\begin{array}{l} Cohen^{\prime }s{\;d}_{F}\left(f\right)=\frac{{\bar{x}}_{S}-{\bar{x}}_{B}}{\sigma_{SB}}, \end{array}$$

where F is the stimulation frequency, *f* ∈ [1, 128], ${\bar{x}}_{S}$ is the mean FFT spectrum over all stimulation epochs of a condition, ${\bar{x}}_{B}$ is the mean over all baseline epochs of that condition, and σ_*SB*_ is the pooled standard deviation over all baseline and stimulation epochs for that condition.


(4) $$\begin{array}{l} bestH_{F}=\underset{h\in \left[1,10\right]}{\mathrm{argmax}}Cohen^{\prime }s{\;d}_{F}\left(h\times F\right), \end{array}$$

where *F* is the stimulation frequency, and *h* represents all available harmonic components for that frequency, up to the 10th harmonic but below the Nyquist frequency (128 Hz).

*Cohen*′*sd* analysis also allowed us to identify the best channel for our exploration. Examining the cortical sites where *Cohen*′*sd* peaked (see Figure 3D), we observed activation in parieto-occipital areas with channel Pz identified as the best (83%) or the second best (17%) for all conditions. This is consistent with our previous work (Tsoneva et al., 2021), where we investigated the cortical sources involved in SSVEP generation, and supports our choice of channel Pz for the consecutive analysis.

*Z*-*score* (see Equation 5) is a standardized score that indicates how far an observation is from a mean of a group of values, the baseline in our case. The *z*-*score* value at the best frequency-specific harmonic component (*bestH*_*F*_) identified above was used for estimating the electrophysiological flicker detection threshold. We selected a cutoff of 0 as the criterion of a trial with detectable SSVEP response (see Equation 6). This cutoff ensured that, in the worse case scenario, an observation would be higher than at least 50% of the baseline epochs.


(5) $$\begin{array}{l} z\text{-}score_{i}\left(f\right)=\frac{x_{Si}-{\bar{x}}_{B}}{\sigma_{B}}, \end{array}$$

where *f* ∈ [1, 128], *x*_*Si*_ is the FFT frequency spectrum of the *i*^*th*^ stimulation epoch of that condition, ${\bar{x}}_{B}$ is the mean over all baseline epochs of that condition and σ_*B*_ is the standard deviation over all baseline epochs for that condition.


(6) $$SSVEP\;response_{i}=\left\{\begin{array}{l} 1,\text{ if }z\text{-}score_{i}(bestH_{F}\times F)>0\text{ (detectable) }\\ 0,\text{ if }z\text{-}score_{i}(bestH_{F}\times F)\leq 0\text{ (not detectable)} \end{array}\right\}$$

where *i* is the trial number, F is the stimulation frequency, and *bestH*_*F*_ is the harmonic component identified in Equation 4.

The percentage of trials resulting in a detectable *SSVEP response* was calculated for each condition (F and MD combination).

#### 2.6.3. Flicker visibility measure

The flicker visibility measure (FVM) was developed by Perz (2019) to predict the flicker visibility of LED light sources by a weighted summation of the relative energy of the frequency components of the light waveform. It is defined as a Minkowski summation of the modulation of each frequency component, normalized by the modulation threshold of a sine or a square wave at the corresponding frequency (see Equation 7).


(7) $$FVM=\sqrt[{2}]{\sum_{m=1}^{\infty }{\left(\frac{C_{m}}{T_{m}}\right)}^{2}}\left\{\begin{array}{l} <1,\text{ not visible}\\ =1,\text{ just visible}\\ >1,\text{ visible} \end{array}\right\},$$

where *C*_*m*_ is the amplitude of the *m*^*th*^ Fourier component of the light waveform divided by the DC value of the waveform, *T*_*m*_ is the visibility threshold of a square waveform at the respective frequency according to the TCSC estimated by Perz (2019).

Because some stimulation frequencies have more MDs that fall under the perception threshold than others, and this might obscure the actual response, for the Cohen's d analysis we only used the trials expected to result in a conscious percept of flicker. We used the FVM as defined in Equation 7 to predict flicker visibility because not all our experiments had subjective responses recorded. The existing subjective report data from the last three experiments and the respective FVM for all conditions were, however, strongly correlated (Pearson correlation ρ = 0.78, *p* < 8.7965*e*−19).

#### 2.6.4. Psychometric function

The psychometric function relates the probability of detection to a specific feature of the stimulus, the MD in our case. It is a specific application of the generalized linear model (GLM) to psychophysical data. To determine the lowest MD necessary to result in detectable stimuli (psychophysically or electophysiologically), i.e. the detection threshold, we use the GLM with a probit link function. The GLM model links the detection probabilities to different MDs for each frequency. The detection probability is the fraction of trials for which a participant subjectively reported that they perceived flicker or the fraction of trials for which the *SSVEPresponse*, as estimated from Equation 6, was detectable. Once a psychometric function was fit to the data, we selected the MDs for which the stimulus was detected with a probability of 50 % (Figure 5).


(8) $$\begin{array}{l} probit\left(p\right)={\text{Φ}}^{-1}\left(p\right)\;\text{for }p\in \left(0,1\right), \end{array}$$

where *p* is the detection probability, and Φ^−1^(*p*) is the inverse of the cumulative distribution function of the standard normal distribution.

We conducted additional assessments of each psychometric fit by performing the deviance test (McCullagh, 2019). Deviance is a generalization of the residual sum of squares, and it measures the goodness of fit compared to a saturated model. Only fits with deviance below one were included in the subsequent analysis.

## 3. Results

### 3.1. Electrophysiological responses

**Figure 2:**
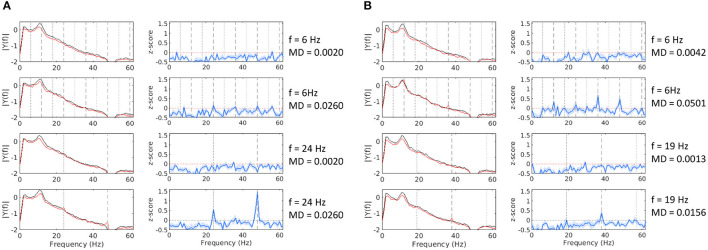
Mean FFT amplitude spectrum and *z*-*score* estimates at channel Pz for 8 conditions from Experiment 2 **(A)** and Experiment 4 **(B)**. The black line denotes baseline epochs, the red line denotes stimulation epochs, and the blue line is the *z*-*score* estimate. Notable frequency components are marked as follows: fundamental (black dotted line), even harmonics (gray dashed lines), odd harmonics (gray dotted line).

For each of the four experiments, we separately analyzed the SSVEP response in all tested conditions. To do so, we first computed the FFT amplitude spectrum for all baseline and stimulation epochs for each condition (i.e., frequency and MD pairs), as described in Section 2.6. Owing to the typical shape of the EEG frequency spectrum and our choice of MDs, which lie extremely low, near perceptual fusion, distinguishing a clear response peak at the fundamental or any harmonic frequency is often difficult. To estimate if a trial resulted in a detectable SSVEP response, we calculated the *z*-*score* of the current stimulation epoch by comparing it to the distribution of all baseline epochs of that condition (see Equation 5). Figure 2 demonstrates eight stimulation conditions from two of our experiments. We could see that the response peaks are more distinguishable in the *z*-*score* dimension, and given a sufficiently high MDs, we can observe the characteristic peaks at the fundamental and harmonic frequencies.

Harmonic components often showed a higher amplitude than the fundamental frequency, especially at frequencies below 20 Hz. Thus, taking the fundamental frequency alone or a summation over the available harmonic components would sometimes mask an otherwise clear response. To avoid this, for each stimulation frequency, we identified the harmonic component, which differentiates best between baseline and stimulation epochs, using the *Cohen*′*sd* measure (see Equation 3). To avoid low MD biases, for this analysis, we only used trials that were expected to result in a visible flicker, as defined by the FVM (see Equation 7).

**Figure 3:**
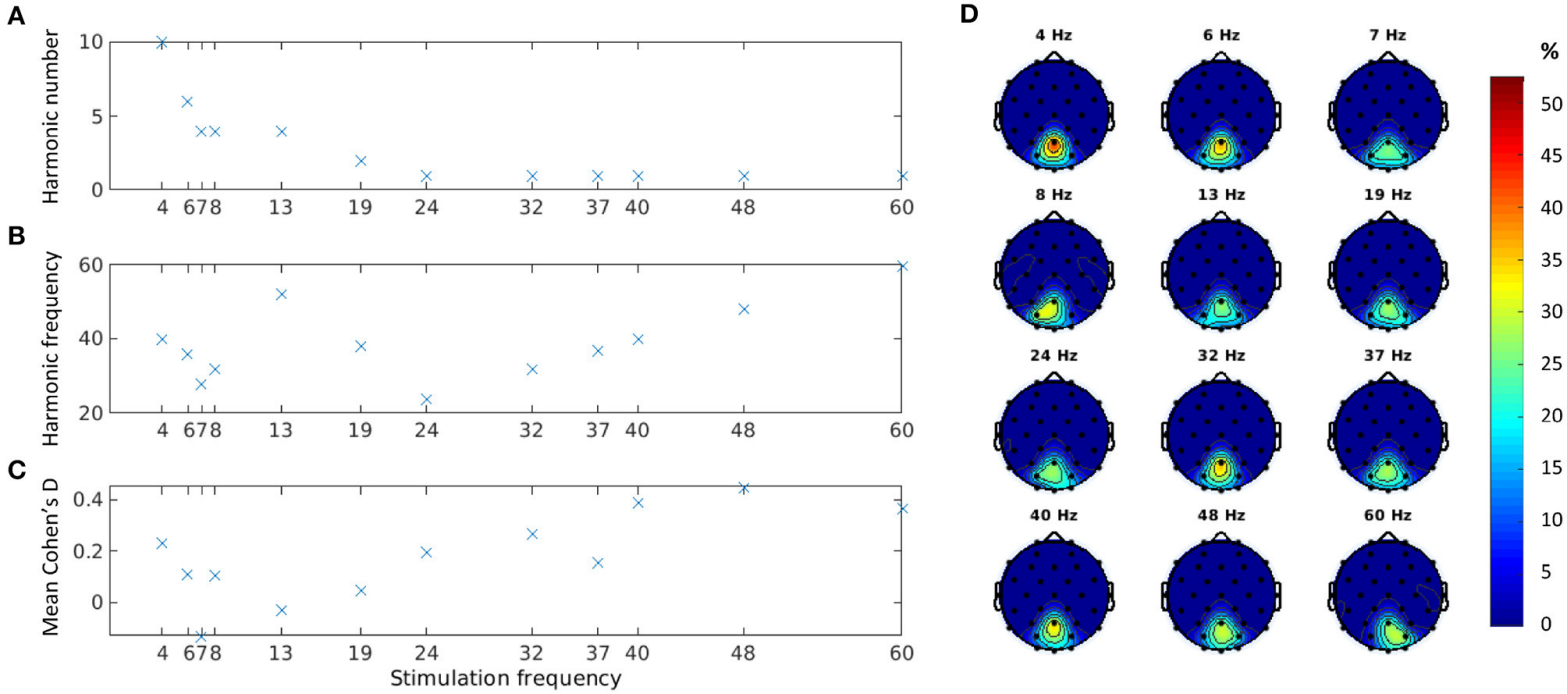
*Cohen*′*sd* estimates for all visible trials at channel Pz: best harmonic component **(A)**; absolute frequency of the best harmonic component **(B)**; and mean *Cohen*′*sd* at the best harmonic component **(C)**. Distribution of location of the best harmonic component over parieto-occipital channels in percentage **(D)**.

Figure 3A shows the harmonic component that produced the best separation between baseline and stimulation epochs of visible trials according to the *Cohen*′*sd* measure (see Equation 4). As observed earlier, for lower stimulation frequencies, this harmonic component was not the fundamental frequency. Indeed, we found that as the stimulation frequency decreased, the harmonic component increased (Pearson correlation ρ = −0.75, p = 0.005). For higher stimulation frequencies (above 20 Hz), the best response was observed at the fundamental component. This analysis identified only even harmonics.

The absolute frequencies of all selected harmonic components seemed to fall in the gamma range (Figure 3B). The *Cohen*′*sd* values, however, showed a small to medium effect size (Figure 3C), possibly due to the extremely low MDs used in the experiments. The three stimulation frequencies with the lowest MDs tested (7, 13, and 19 Hz in Experiment 3) indeed showed a particularly low *Cohen*′*sd* effect size on average.

To assess the validity of our estimations and ensure that the identified peaks correspond to the expected SSVEP response frequencies, we conducted further analysis by examining the distribution of maximum *Cohen*′*sd* values over the whole frequency spectrum. For all frequencies, except 7 Hz, the maximum *Cohen*′*sd* values were, concentrated at the expected harmonic frequencies or the fundamental frequency. Higher frequencies (≥19*Hz*) had a distinct single peak at the fundamental frequency, and lower frequencies could have multiple peaks (e.g., at two different harmonic frequencies). In contrast, the 7-Hz condition showed a uniformly distributed pattern of maximum *Cohen*′*sd* values that did not align with the expected SSVEP response frequencies. Based on these findings and the generally extremely low *Cohen*′*sd* value at 7 Hz discussed above, we decided to not use the data from this condition in the contrast sensitivity estimation.

For each harmonic component, we also explored the cortical sites where *Cohen*′*sd* peaked. Figure 3D shows the parieto-occipital channels with the highest *Cohen*′*sd* for the selected best harmonic across subjects for each stimulation frequency. Channel Pz scored the best for 10 out of 12 stimulation frequencies and was second, just a few hundredths short of the best channel, for the other two.

**Figure 4:**
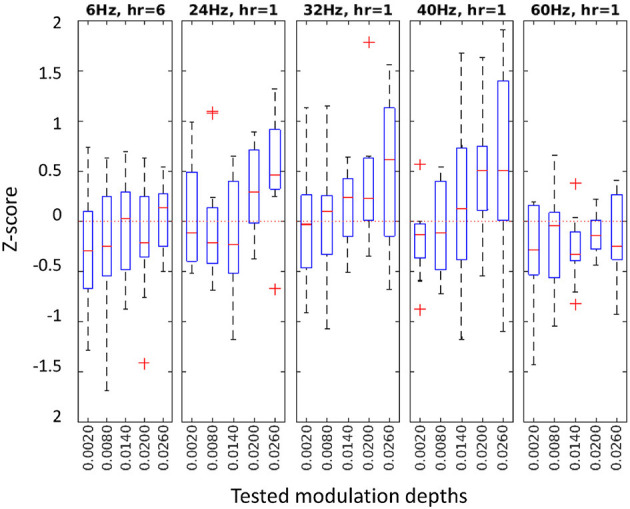
*z*-*score* distributions at the best harmonic component for each condition in Experiment 2. Different frequencies with their selected harmonic component (*hr*) in separate panels, with tested MDs on the horizontal axes.

Our flicker detection criterion for a stimulation frequency was, then, the *z*-*score* at the best harmonic component (see Equation 6) at channel Pz. Figure 4 shows the *z*-*score* distributions for each tested condition in Experiment 2. See Supplementary material for the results of the other three experiments.

The mean *z*-*score* of all detectable conditions was 0.67 (SD: 0.76), ensuring that at least 75% of the baseline values were below the RVS response. A significant low to moderate correlation was observed between the MD and the *z*-*score* (Pearson correlation ρ = 0.31, *p* < 0.0001) across all frequencies and MD conditions.

### 3.2. Temporal contrast sensitivity function

**Figure 5:**
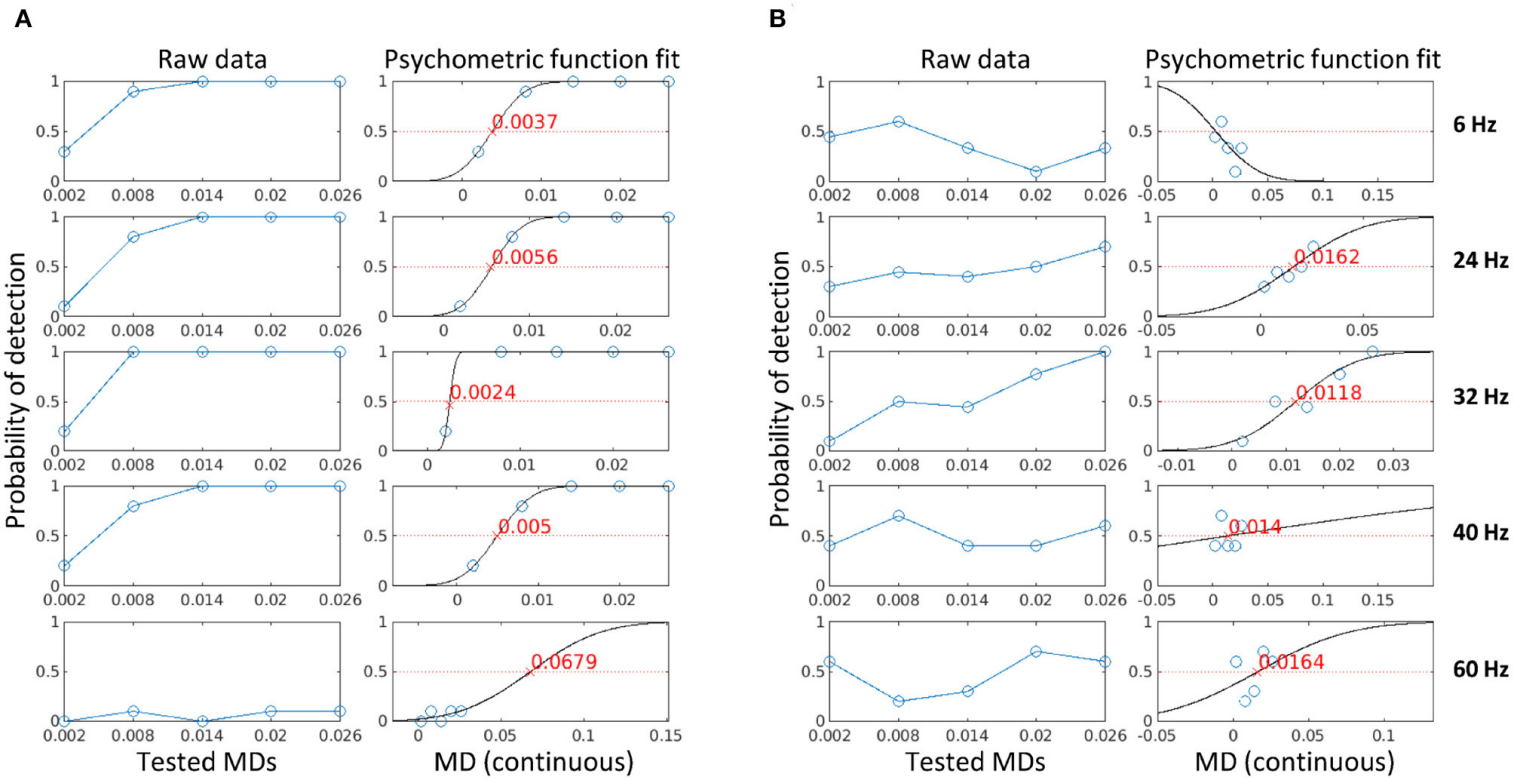
The psychometric curve fit using psychophysical data **(A)** and electrophysiological data **(B)** for one participant in Experiment 2. The blue circles are the probability of detection for each condition (stimulation frequencies in separate panels, MD on the horizontal axes), the black line is the psychometric curve fit, and the red cross is the visibility threshold corresponding to the MD for which flicker can be detected with a probability of 50%.

To find the visibility threshold of a stimulation frequency, we fitted a psychometric function to the probability of detection at each MD for each subject, as described in Section 2.6. The probability of detection is defined as the fraction of trials where a flicker is detected using either the subjective reports collected in the three later experiments (i.e., psychophysical data) or the *z*-*score* estimates using Equation 6 (i.e., electrophysiological data). Figure 5 shows the calculated probability of detection (columns 1 and 3) and the psychometric curve fit (columns 2 and 4) for one subject in Experiment 2 for psychophysical data (A) and electrophysiological data (B). The visibility threshold is defined as the modulation depth for which flicker can be detected with a probability of 50% (the red crosses in the figure). If the fit had a negative slope, the estimated visibility threshold was negative, or if the psychometric fit deviance exceeded one, then no estimate could be provided for this condition (see 6 Hz in column 4 of the figure). As expected, the psychophysical data showed less variability and a closer fit as compared to the electrophysiological data. However, for all conditions, both psychophysical and electrophysiological visibility thresholds could be estimated for the majority of participants. Depending on the stimulation frequency and the different number of participants in each experiment, this resulted in 10–30 data points per frequency for psychophysical data and 9–30 data points per frequency for electrophysiological data.

**Figure 6:**
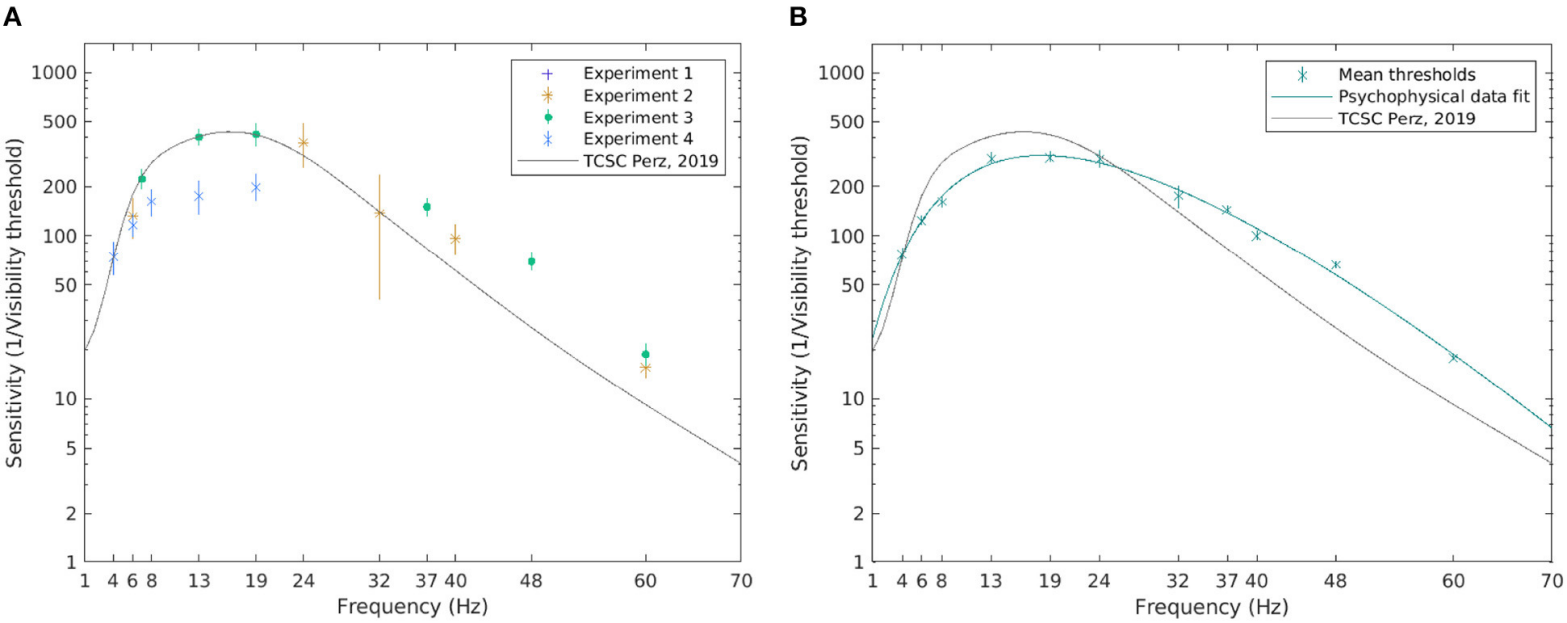
The visibility thresholds and the TCSC derived from psychophysical data. The median of the visibility thresholds over subjects in each of the experiments; error bars depict the 95% CI **(A)**. The TCSC estimated as a rational polynomial fit over the mean of the visibility thresholds for all subjects exposed to the condition; error bars depict standard error **(B)**. Note that a logarithmic scale is used on the vertical axis.

Temporal contrast sensitivity is defined as the reciprocal of the modulation depth at the visibility threshold. Figure 6 shows the inverse of the visibility thresholds and the TCSC derived from the psychophysical data. The estimated sensitivity for all conditions in each experiment appears to fall close to the TCSC of Perz (2019) (see median and the 95% confidence intervals (CI) in Figure 6A).

To estimate the TCSC across all experiments, we pooled the sensitivity estimates for all subjects exposed to a certain frequency, excluding outliers deviating more than two standard deviations from the mean. A cubic/linear rational polynomial, such as the one defined in Equation 10, using the Levenberg-Marquardt algorithm (Marquardt, 1963), was fitted over the mean values. The estimated coefficients with their 95% CIs are listed in Table 3. To assess the goodness of fit, the coefficient of determination, *R*^2^, between the contrast threshold function and mean visibility thresholds was calculated, yielding *R*^2^ = 0.99, indicating that 99% of the variability in the mean visibility threshold is explained by frequency. Figure 6B shows the resulting TCSC and how it compares to that obtained by Perz (2019). We see that the estimated sensitivity function increases with frequency for frequencies up to 10 Hz, peaks around 18 Hz (reaching a value of 309), and decreases thereafter.

**Table 3:** Psychophysical data fit coefficients.

**Name**	**Coefficients**	**95% CI**
*p*_1_	-0.0008	(-0.008, 0.006)
*p*_2_	-0.409	(-1.283, 0.466)
*p*_3_	40.68	(14.44, 66.92)
*p*_4_	121.50	(-225.20, 468.10)
*q*_1_	10.75	(-11.98, 33.49)

**Figure 7:**
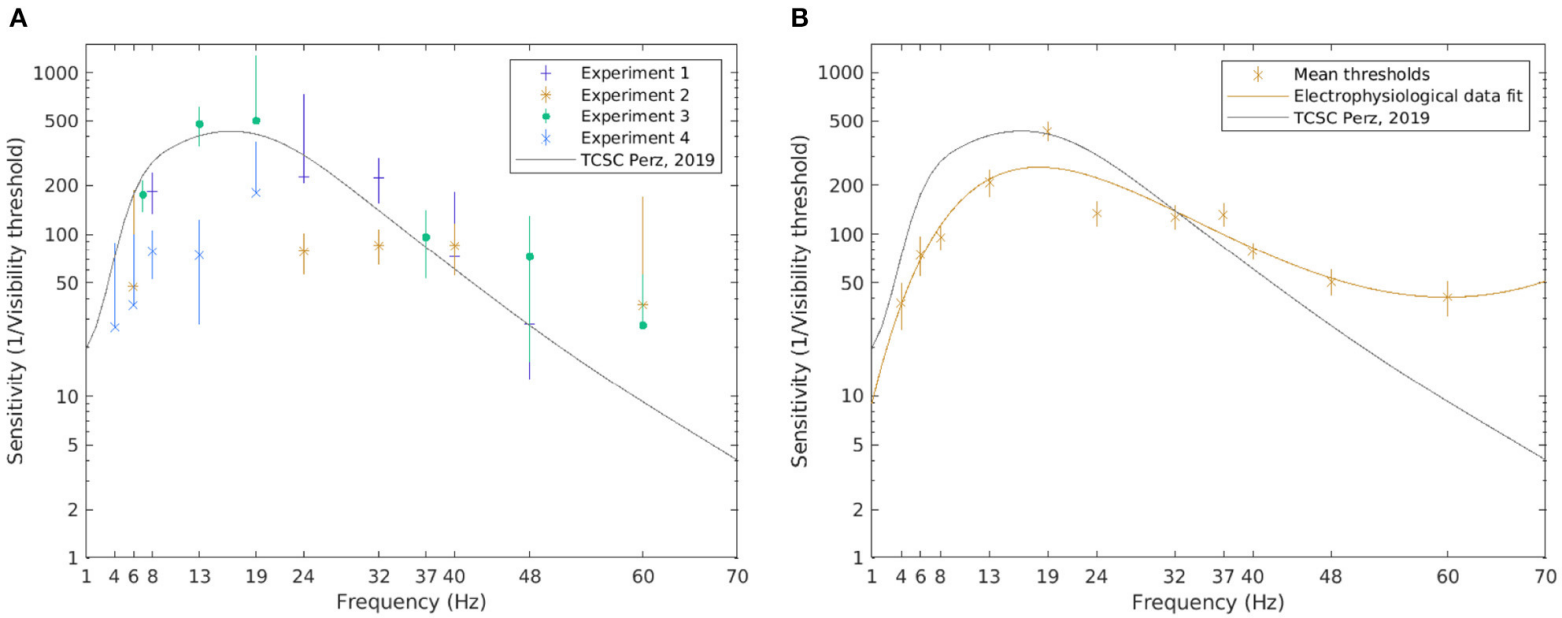
The visibility thresholds and the TCSC derived from electrophysiological data. The median of the visibility thresholds over subjects in each of the experiments; error bars depict the 95% CI **(A)**. The TCSC estimated as a rational polynomial fit over the mean of the visibility thresholds for all subjects exposed to the condition; error bars depict standard error **(B)**. Note that a logarithmic scale is used on the vertical axis.

The results for electrophysiological data are shown in Figure 7. The same procedure as with psychophysical data was followed for estimating the TCSC based on *z*-*score* values. A cubic/linear rational polynomial such as the one defined in Equation 10 was fitted over the mean values for all subjects exposed to a certain frequency, excluding outliers. The resulting coefficients with their 95% CIs are listed in Table 4. The estimated *R*^2^ statistics was 0.88, indicating that, according to our model, 88% of the variability in the mean visibility thresholds was explained by frequency.


(9) $$\begin{array}{l} TCSC\left(f\right)=\frac{\left(p_{1}*x^{3}+p_{2}*x^{2}+p_{3}*x+p_{4}\right)}{\left(x+q_{1}\right)} \end{array}$$

**Table 4:** Electrophysiological data fit coefficients.

**Name**	**Coefficients**	**95% CI**
*p*_1_	0.014	(-0.033, 0.060)
*p*_2_	-1.69	(-6.78, 3.41)
*p*_3_	71.75	(-76.35, 219.80)
*p*_4_	155.90	(-851.10, 1163)
*q*_1_	22.65	(-70.55, 115.90)

Generally, more variability is observed in the visibility threshold estimates based on electrophysiological data (Figure 7A). Some differences between separate experiments could also be noted. The resulting TCSC based on electrophysiological data shown in Figure 7B, however, resembles the shape of the psychophysiological curve. Although it starts at a lower point, for frequencies below 10 Hz, the sensitivity function increases with frequency. It peaks at 18 Hz (reaching a value of 258) and decreases for higher frequencies but at a slower rate compared to the psychophysical fit.

**Figure 8:**
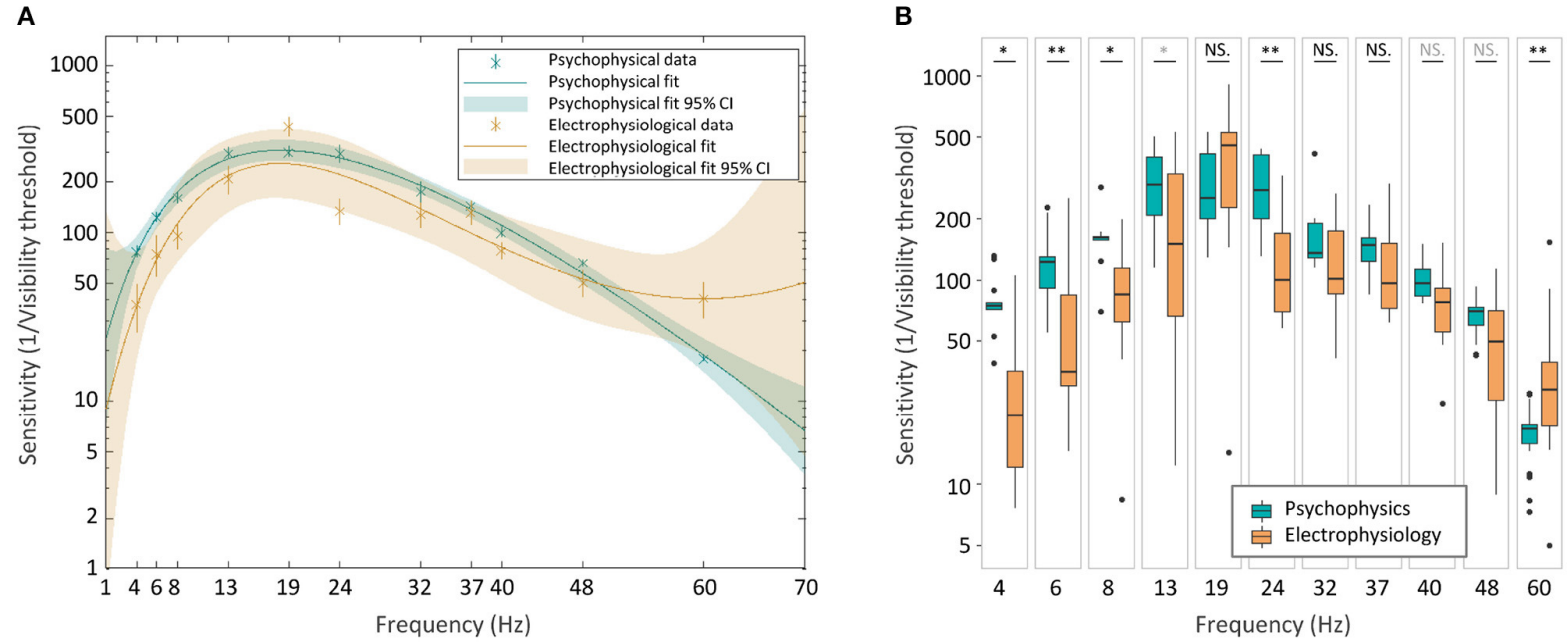
Psychophysical and electrophysiological TCSCs comparison. The two curves with their 95% confidence prediction bounds **(A)**. Boxplots of sensitivity values **(B)**. The statistical significance of the difference between the two groups was assessed using a two-sided Wilcoxon rank sum test (*p < 0.05, **p < 0.01, in gray *q* < 0.005, in black *q* < 0.001).

To further assess the two TCSCs, we calculated the prediction bounds for the fitted functions at the 95% confidence level (Figure 8A). These bounds reflect the variability associated with the fitted curves' estimates. Although there is considerable overlap between the psychophysical and electrophysiological curves, both sets of prediction bounds consistently deviate from zero. This suggests a significant non-zero effect, and together with the high coefficients of determination, it provides evidence that the fitted curves capture a systematic relationship between frequency and flicker sensitivity.

To investigate potential differences between the curves, we conducted a non-parametric two-sided Wilcoxon rank sum test (Mann and Whitney, 1947), comparing the sensitivity estimates for psychophysical and electrophysiological measures at each frequency. The analysis revealed significant differences at frequencies below 19 Hz and at 24 Hz and 60 Hz (Figure 8B), suggesting potentially distinct characteristics of the two curves at different frequencies. To account for multiple comparisons, we used the false discovery rate test (Storey, 2002), which is a statistical measure that controls the proportion of false positives amongst the significant results when performing multiple hypothesis testing. The estimated *q*-values ranged between 0 and 0.005 confirming the significance of the results.

## 4. Discussion

In four experiments, we built a temporal contrast sensitivity model based on the electrophysiological responses to RVS and compared it to psychophysical models of flicker visibility. Data from 62 observers revealed that the resulting electrophysiological TCSC had a similar shape to the psychophysical counterpart, with maximal sensitivity at 18 Hz. However, differences were observed at lower frequencies and in the gamma range, indicating potential disparities between the cortical and perceptual mechanisms that apply at lower and higher temporal frequencies. Our results suggest that the SSVEP response can be measured even without the conscious perception of a flicker, particularly at frequencies above 50 Hz. The presence of even harmonics in the response provides evidence of non-linear processing in the visual system.

### 4.1. Temporal contrast sensitivity functions

The observed differences between the electrophysiological and psychophysical sensitivity curves generally align with previous literature findings. Previous studies have also reported a less steep decline in electrophysiological sensitivity as compared to the psychophysical estimates at high frequencies (van der Tweel, 1964; van der Tweel and Verduyn Lunel, 1965; Sokol and Riggs, 1971). Possible explanations for this disparity can be sought at multiple levels of the visual system, taking into account various contributing factors.

The electrical responses at high frequencies have extremely short latencies, which are assumed to precede the flicker decision stage and can be considered to be of a primary origin. van der Tweel and Verduyn Lunel (1965) reported latencies of 250-300 ms for stimuli of approximately 10 Hz, as compared to 30 to 65 ms for frequencies above 35 Hz (Regan, 1989; Tsoneva et al., 2021). This means that some supplementary attenuation might occur before conscious flicker perception (van der Tweel and Verduyn Lunel, 1965).

Low frequency flicker of 1 Hz to 10 Hz is more readily perceived in foveal than in peripheral vision, whereas, at frequencies above 30 Hz, this is reversed (McKee and Taylor, 1984; Perz, 2019). Already in the early studies, Robson (1966) argued that the fall-off in sensitivity at low temporal frequencies is the result of antagonism between signals from the center and surround regions of the receptive fields. Peripheral retinal cells tend to have higher contrast sensitivity and respond at higher flicker frequencies than those closer to the fovea, and in fMRI studies, contrast sensitivity at approximately 20 Hz flicker has been found to increase in peripheral voxels when compared to foveal voxels (Himmelberg and Wade, 2019). Temporal response properties of peripheral retinal cells appear to be predominantly represented in the cortical periphery of early visual areas (V1, V2, V3, and V3a). This sensitivity bias, also present at the retinal (Sinha et al., 2017) and lateral geniculate nucleus (LGN) (Connolly and Van Essen, 1984; Krolak-Salmon et al., 2003) levels, might be compensated later in the cortical visual pathway, in hV4, and, possibly, in other higher-order ventral regions, where temporal sensitivity is more similar to the psychophysical estimates (Himmelberg and Wade, 2019). Other authors have also attributed medium- and high-frequency components of SSVEPs to sources in the primary visual cortex (van Dijk and Spekreijse, 1990), while low-frequency components of SSVEPs may be generated not only by cortical regions but also by subcortical structures, such as the retinal level or in fiber tracts (Spekreijse et al., 1977). The large visual field used in our experiments would have undoubtedly stimulated both central and peripheral vision cells at the retinal and (sub)cortical levels, which might trigger different visual pathways depending on frequency.

SSVEP responses are, indeed, sensitive to cortical resonances in at least three frequency subsystems (Regan, 1977; Srinivasan et al., 2006): “low-frequency” response (1–12 Hz) peaking around 10 Hz, a “medium-frequency” response (13–25 Hz) which peaks around 18 Hz, and a “high-frequency” response produced by RVS of approximately 30–60 Hz. SSVEP responses in these different frequency bands show different sensitivities to the physical parameters of the flicker, suggesting that they might be engaging functionally distinct, although spatially overlapping, cortical networks (Regan, 1989; Ding et al., 2006). Vialatte et al. (2009) argued that the increase in synchrony of the oscillatory events during stimulation is suggestive of a reorganization of the oscillatory patterns in the visual system by the flickering stimulation, which propagates preferentially through the visual pathway depending on frequency. They found that the 32 Hz stimulation preferentially activates the magnocellular pathway while the 16 Hz stimulation activates the parvocellular pathway. Such frequency-dependent preferential activation has also been reported in the context of attention, where attention to a flicker stimulus increases or decreases SSVEP amplitude and phase locking depending on which of the two cortical networks were selected (Ding et al., 2006).

The differences at frequencies below 50 Hz might also be due to some cross-frequency interactions with spontaneous neural activity. Stimulus response and ongoing oscillatory activity appear to be additive to a large degree, and the responses could be diminished by decreasing the modulation depth so that the ongoing activity prevails (van der Tweel and Verduyn Lunel, 1965; Norcia et al., 2015). Event-related alpha desynchronization during visual processes has been previously reported (Pfurtscheller et al., 1994; Suffczynski et al., 2001), which might have implications for the responses in that range. Variation in evoked responses to flicker across sources of endogenous alpha oscillatory activity has also been observed (Nuttall et al., 2022). In our previous research (Tsoneva et al., 2015), we also noted several distinct evoked patterns in delta (1–4 Hz), theta (4–8 Hz), alpha (8-12 Hz) and beta (15-30 Hz) bands, which could contribute to the SSVEP response measured at those frequencies. The higher variability in the responses at low frequencies but not at high frequencies (predominantly in the gamma band) observed in our experiment and reported in the literature (van der Tweel and Verduyn Lunel, 1965; Sternheim and Cavonius, 1972; Wei et al., 2018) might be a result of these interactions.

The inter-subject variability of the electrophysiological thresholds we observed was higher than that of the psychophysical estimates, which was also reported in other experiments (van der Tweel and Verduyn Lunel, 1965; Sternheim and Cavonius, 1972; Wei et al., 2018). Selective properties of the brain structures of some subjects, at frequencies in the alpha band, for example, could explain part of the variability (van der Tweel, 1964). Furthermore, physically similar stimuli can elicit different electrical responses depending upon their significance to the subject (Chapman and Bragdon, 1964) or different conscious experiences altogether (Carmel et al., 2006).

We also noted some differences between the psychophysical TCSC measured in our experiment and the one used as a reference (Perz, 2019), which were obtained using the same physical setup. This might be a result of the difference in average luminance and experimental paradigm. Perz (2019) used an average luminance level, the amount of light reflected by a surface, of 209*cd*/*m*^2^ and the average illuminance, the amount of light falling onto a surface, of 500 lx, while in our experiments, we used an average luminance level of 418*cd*/*m*^2^ and illuminance of 1000 lx. Generally, the difference between curves measured at different background illuminance levels becomes smaller the further away we go from the scotopic vision region (de Lange, 1958; Kelly, 1961). At relatively high background illuminance levels (>80*cd*/*m*^2^), this difference is particularly small. Perz (2019) also used the staircase procedure (Cornsweet, 1962), while we used a detection task. The advantage of the staircase method is that it requires the presentation of fewer stimuli than any other psychophysical method because, after the first few stimuli, all others are close to the threshold level. To increase the SNR of EEG signals, however, we need multiple trials per condition. Averaging over 10 trials per condition improves the SNR by a factor of $\sqrt{\left(10\right)}\approx 3$ (Andreassi, 2007). Furthermore, in an online procedure, such as the staircase method, the criterion of detection should be known beforehand, which was not the case here. We sampled our conditions around the expected threshold levels to make sure that the sensitivity point could be included. We also conducted multiple experiments employing the same frequencies if we were uncertain that the electrophysiological threshold was within the modulation boundaries we have selected previously.

### 4.2. Harmonic responses and non-linearities

For most of our stimulation frequencies, the absolute frequency of the best harmonic component lied in the gamma band (30–60 Hz). Internal oscillations in the gamma range, especially around 40 Hz, have been repeatedly mentioned in relation to sensory processing, attention selection, and working memory maintenance (Başar-Eroglu et al., 1996; Jensen et al., 2007). Gamma oscillations have been associated with the binding problem and conscious perception, possibly through temporal synchrony across distinct brain regions (Buzsáki, 2009). The synchronization of neuronal discharges can serve for the integration of distributed neurons into cell assemblies, and this process may underlie the selection of perceptually and behaviorally relevant information (Engel et al., 1999).

We also observed that the harmonic component that separates best between stimulation and baseline epochs is different for low as compared to high frequencies. For frequencies below 20 Hz, this was not the first harmonic component. Importantly, the lower the frequency, the higher the harmonic component identified (Pearson correlation ρ = −0.75, *p* = 0.005). The decreasing SNR trend of EEG with increasing frequency and the limited number of harmonics available at high frequencies could have played a role here, but the consistent declining trend is, anyway, quite notable in Figure 3A. Previous studies have also reported that, at lower frequencies, the second harmonics are larger in amplitude than the fundamental (van der Tweel, 1964; van der Tweel and Verduyn Lunel, 1965; Regan, 1968; Fiorentini and Trimarchi, 1992). Additionally, unlike our previous studies using high-frequency stimulation (40–60 Hz) (Tsoneva et al., 2015, 2021), in the current study, we did not observe sub-harmonic responses. Generally, the SNR of sub-harmonic responses was lower compared to higher harmonic components, and at extremely low MDs, such as the ones used here, it might be difficult to dissociate sub-harmonics from ongoing oscillatory activity.

In a linear system, the response should have been confined to the frequencies present in a square waveform, namely the fundamental and the odd harmonic components. The presence of even harmonics and the fact that they show a better response for many frequencies suggest non-linear processing in the visual system. Although we cannot provide an exact explanation for the occurrence of even harmonics, we can speculate on the potential factors that may have contributed to them. Watson et al. (1986) argued that many non-linear elements figure in models of temporal sensitivity, and they can be categorized into three types: *output non-linearities* lying between the internal and psychophysical response, *adaptive processes* altering system processes with a change in the adaptive state, and when signals pass through *different pathways*, such as a transient and sustained pathway. We will briefly discuss the first two concepts, as they are more relevant to our findings.

The presence of cortical resonance frequencies, leading to preferential amplifications of certain frequencies in the SSVEP response (Regan, 1977; Herrmann, 2001), preferential network tuning, triggering different visual pathways depending on frequency (Vialatte et al., 2009; Himmelberg and Wade, 2019), and interactions with ongoing neural activity, such as the spontaneous sensory sampling modulated by pre-stimulus theta (4–7 Hz) and alpha (8–14 Hz) band phases (Mathewson et al., 2009; Haegens and Golumbic, 2018), could contribute to the *output non-linearities*. Such kind of non-linearities are more likely to occur at an SNR that is not too small (as in high frequencies or extremely low MDs) because noise will often act as a linearizing factor (van der Tweel and Verduyn Lunel, 1965). Consequently, these effects may be more pronounced at low frequencies and higher modulation depths, similar to what we observed.

*Adaptation* could also introduce non-linearities. After adaptation to a more frequent stimulus, the responses of the individually tuned neurons to that stimulus from the population are reduced, resulting in an imbalance in the overall response to the periodic stimulus (Norcia et al., 2015). In our case, the constant luminance in the inter-stimuli-interval would have been the more frequent stimuli, thus, resulting in some adaptation to the non-flickering stimuli. Alternatively, long stimuli presentation could result in adaptation, characterized by an initial increase and a subsequent decline in SSVEP amplitude, which would also introduce a non-linearity. However, this effect usually occurs with longer exposure, typically in the range of 10 s, but some inter-subject variability might be present (Ho and Berkley, 1988; Labecki et al., 2019).

### 4.3. Limitations and biases in temporal sensitivity assessment

Temporal sensitivity is affected by various factors such as background intensity, pupil variations, adaptation, stimulus size and the methods by which the visibility thresholds are obtained.

Background light intensity, pupil size, and retinal illuminance play an important role in temporal sensitivity. The estimated retinal illuminance in our experiment varies, depending on pupil size, from 1,300 Td for a constricted pupil to an unlikely 21,000 Td for a dilated pupil (Watson and Yellott, 2012). According to multiple models (Watson and Yellott, 2012), at an illuminance level of approximately 400*cd*/*m*^2^, the expected pupil diameter is consistently estimated to be 2.5 mm, which corresponds to a retinal illuminance of 2,000 Td. Given that there is minimal difference in the temporal sensitivity curves at high retinal illuminance levels (above 1,000 Td), as mentioned earlier (Watson et al., 1986), we can reasonably assume that these factors will not have a significant impact on our estimated curve.

Flicker adaptation can lead to reduced sensitivity to flicker and changes in neural processing. Sensitivity to temporally modulated light is known to be lower after adaptation to a flickering light than after adaptation to a steady light of the same time-averaged retinal illuminance (Kelly, 1972). To prevent flicker adaptation, similar to the experimental procedure used in Perz (2019), light at a constant luminance was presented after each stimulus for a random interval of at least 3 seconds, and the various stimuli for all lighting conditions were intermingled and presented in a random order, different per participant. Although we controlled for flicker adaptation within an experiment, some variations in flicker adaptation may have occurred between experiments. For instance, Experiment 4 focused on low frequencies with relatively high MDs, while Experiment 2 included many low-frequency conditions with extremely low MDs. These differences may have influenced the sensitivity obtained in the experiments at a population level (see Figures 6A, 7A), contributing to the variability in the final threshold estimates.

Further, conditions with higher MDs may have approached the levels at which the SSVEP responses saturate, potentially affecting the electrophysiological visibility thresholds. According to Regan (1989), a gradual increase of MD at a constant mean luminance level eventually leads to a moment when the amplitude of the low-frequency VEPs (1–13 Hz) reaches a maximum level (saturates) or may even grow lower (over-saturates). To avoid saturation effects, we generally kept the MD below 0.15 in all experiments, which is below the expected saturation levels based on the literature (MD of 0.4 at 5 Hz and MD of 0.3 at 30 Hz for 2 degrees visual field in Regan (1989), p. 383). Although the large visual field and higher harmonic components used in our experiments could have a different saturation range, a visual inspection of the data did not reveal any consistent saturation effects.

Stimulus size effects are characterized by a decrease in low-frequency sensitivity and a small increase in high-frequency sensitivity (Brundrett, 1974; Watson et al., 1986). At high frequencies, flicker perception is typically more pronounced in peripheral vision (Rovamo and Raninen, 1984; Perz, 2019). Often, models using small stimuli presented in central vision will not be able to correctly predict flicker visibility in the periphery. By utilizing a large visual field and high illuminance levels, we ensured that we captured the most sensitive case, and our findings could inform a range of SSVEP applications. Importantly, expected variations in sensitivity are more likely at low frequencies, while the impact on sensitivity estimates at high frequencies, which are becoming more popular in SSVEP research, would be comparatively smaller (Watson et al., 1986; Barten, 1999).

The choice of detection criterion also influences the assessment of contrast sensitivity. Deriving a sensitivity function requires a response that varies monotonically with stimulus modulation. The SSVEP appears to be a linear function of log stimulus contrast for a substantial range of suprathreshold contrasts, starting near the psychophysical threshold (Norcia et al., 2015) and extending to a point when saturation is reached at very high MDs (Sternheim and Cavonius, 1972). This can be seen in Figure 4. Our choice of *detection criterion*, namely the *z*-*score* at the best harmonic component for that frequency with a cutoff of zero, provides a monotonically increasing probability of detection with increasing MDs, where the SSVEP response is expected (e.g. 24, 32 and 40 Hz), and a low probability of detection for MDs, where a detectable SSVEP response is unlikely (e.g., 60 Hz with all MDs below perception), thus satisfying the above requirement. This requirement can also be confirmed by the significant correlation between MD and *z*-*score* (Pearson correlation ρ = 0.31, p < 0.0001) across all frequency and MD conditions. It should be noted, however, that this correlation does not account for potential non-linearities, such as the expected sensitivity differences between frequencies and the decreasing SNR trend of the EEG frequency spectrum with increasing frequency.

While the *z*-*score* at the best harmonic component satisfied the psychometric function requirements, some unexplained inter-individual variability remains, and other detection criteria based on multi-channel data analysis using, for example, common spatial patterns or canonical correlation analysis could also be considered. The *z*-*score* cutoff value can be adjusted to balance sensitivity and variability in the data. Increasing the cutoff value could reduce variability but at the cost of decreasing sensitivity overall. Therefore, the optimal cutoff value should be carefully considered and chosen based on the specific application and research question.

The choice of the model is also important. We aimed to maintain a physiologically plausible fit with an excellent goodness of fit coefficient, even though this resulted in relatively large CIs of coefficients. For linear coefficients, if the bounds cross zero, it indicates that the coefficients may not significantly differ from zero. However, in the case of a rational polynomial fit and logarithmic data, the model becomes inherently non-linear as the predictor variables are raised to negative and/or fractional powers. This makes the interpretation of the coefficients CIs more challenging. A polynomial fit yields significant coefficients but extrapolates the data beyond our measured range in a physiologically unlikely manner (i.e., increasing sensitivity with increasing frequency beyond the maximum at lower frequencies). Therefore, we opted to use the rational fit.

Due to practical constraints, it was not feasible to expose every subject to all conditions, as this would have made the experiment duration too long and introduced unwanted effects such as fatigue and lapses of attention. Therefore, our sensitivity estimates are based on different subsamples at different frequencies. This introduces some inherent limitations in estimating the overall variability and conducting a direct statistical comparison between the curves. Despite these limitations, the statistical analysis we conducted provides valuable indications of potential differences between the psychophysical and electrophysiological measures at various frequencies.

Another methodological bias that needs to be acknowledged is the potential influence of the beep sound that accompanied the stimuli presentation. This could have served as a warning cue and may have engaged subjects' attentional processes, potentially influencing sensitivity thresholds. Spatial selective attention is a well-established phenomenon that can enhance the amplitude of SSVEPs when attention is directed to the stimulus (Morgan et al., 1996; Müller et al., 1998). This specific instruction was given to our study subjects. Although we cannot completely rule out occasional lapses of attention, we expected any such occurrences to be averaged out in the final results. Given the stability of the SSVEP response, any additional effects of sensory or attentional processes (e.g., triggered by cueing), usually measured within the first few milliseconds after stimulus onset, would likely have a negligible impact on the narrow band filtered signal around the stimulation frequency, averaged over 3 seconds. In the case of any residual effects, we expected their impact to be consistently observed across all conditions.

Overall, we aimed to capture the most sensitive case that is applicable to a wide range of SSVEP applications. However, it must be noted that our findings may not automatically be generalized to RVS with inherently different properties, such as patterned stimulation (i.e., gratings or pattern reversal), low luminance level (e.g., below 80*cd*/*m*^2^ or in scotopic vision), smaller visual fields, monocular vision or overt attention paradigms. Further research is required to clarify these matters.

Lastly, we did not aim to determine how temporal sensitivity varies with individual characteristics and external factors. This falls beyond the scope of our research and needs additional research. This study proposes a temporal sensitivity model for a “standard observer,” which can be useful for the current applications of SSVEP in research and clinical practice.

## 5. Conclusions

We demonstrated that an electrophysiological model of temporal contrast sensitivity based on SSVEP responses to full field luminance modulation could be established. The resulting TCSC was similar in shape to its psychophysical counterpart, confirming that the human visual system is most sensitive to RVS at frequencies between 10 and 20 Hz. The electrophysiological sensitivity, however, was lower than the psychophysical TCSF for frequencies below 50 Hz, while above this frequency, an SSVEP response could be measured without conscious flicker perception, with non-linear mechanisms playing a role.

The difference in cortical and perceptual mechanisms that apply at higher and lower stimulation frequencies must be considered in SSVEP applications in research and clinical practice. There might be intrinsic interactions and non-linearities in the processes under investigation, which might make it difficult to dissociate the contribution of different frequency subsystems or separate the explanatory or causal role of oscillations at those frequencies. These considerations are particularly relevant when studying rhythmic brain activity, functional brain connectivity, and pathological brain dynamics using RVS. Our insights can also be helpful for the design of future experiments involving RVS, both by informing the development of more robust experimental paradigms and aiding in the correct interpretation of results. Notably, stimulation at high frequencies presents potential benefits for enhancing patient comfort and safety by reducing the risks of epileptic seizures, headaches, or visual fatigue (Fisher et al., 2005; Makri et al., 2015). For such applications, our results will help to determine the optimum parameter settings for RVS stimulation at the thresholds of visibility. Finally, our findings can also complement the psychophysical research on temporal artifacts for general lighting purposes by defining the boundaries of the physiological effects of flicker.

## Data availability statement

The datasets presented in this article are not readily available because it will potentially compromise research participant consent under the European GDRP law regarding reuse of data for secondary research and development purposes. Requests to access the datasets should be directed to tsvetomira.tsoneva@philips.com.

## Ethics statement

The studies involving human participants were reviewed and approved by Internal Committee Biomedical Experiments (ICBE) at Philips Research Europe. The participants provided their written informed consent to participate in this study.

## Author contributions

TT and GG-M conceived and designed the experiments. TT wrote the article and analyzed the results. PD supervised the work and reviewed the article. All authors contributed to the article and approved the submitted version.
