# Supplementary material for: Electrophysiological model of human temporal contrast sensitivity based on SSVEP
Source: Front Neurosci. 2023 Jul 31;17:1180829. doi: 10.3389/fnins.2023.1180829 (PMC10433170; doi:10.3389/fnins.2023.1180829)
Supplement: Supplementary file 1 [file Data_Sheet_1.PDF]

Supplementary Material

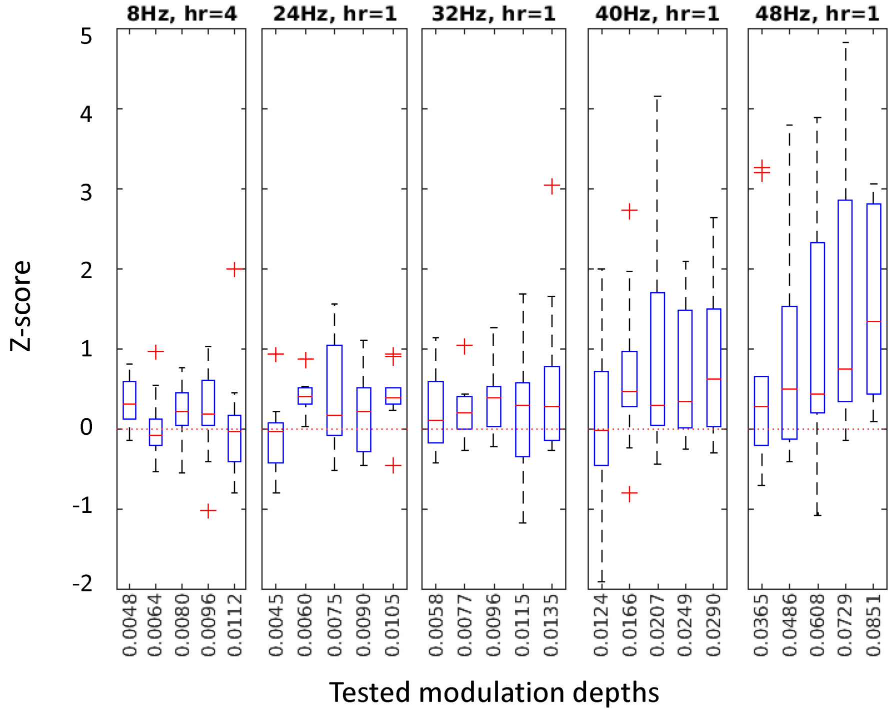

Figure S1. $z\text{-}score$ distributions at the best harmonic component for each condition in Experiment 1. Different frequencies with their selected harmonic component in separate panels, tested MDs on the horizontal axes.

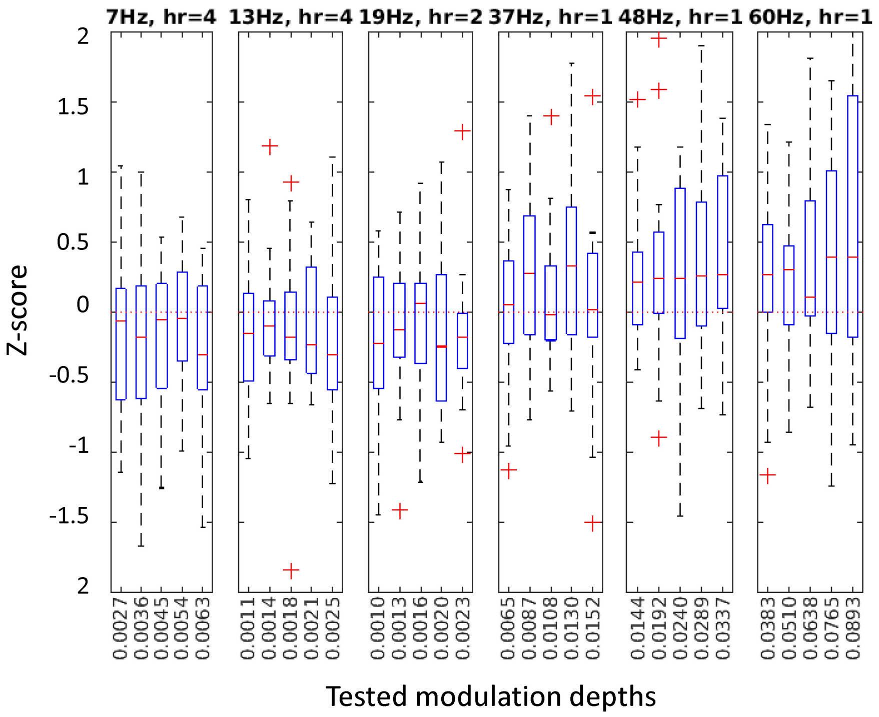

Figure S2. *z-score* distributions at the best harmonic component for each condition in Experiment 3. Different frequencies with their selected harmonic component in separate panels, tested MDs on the horizontal axes.

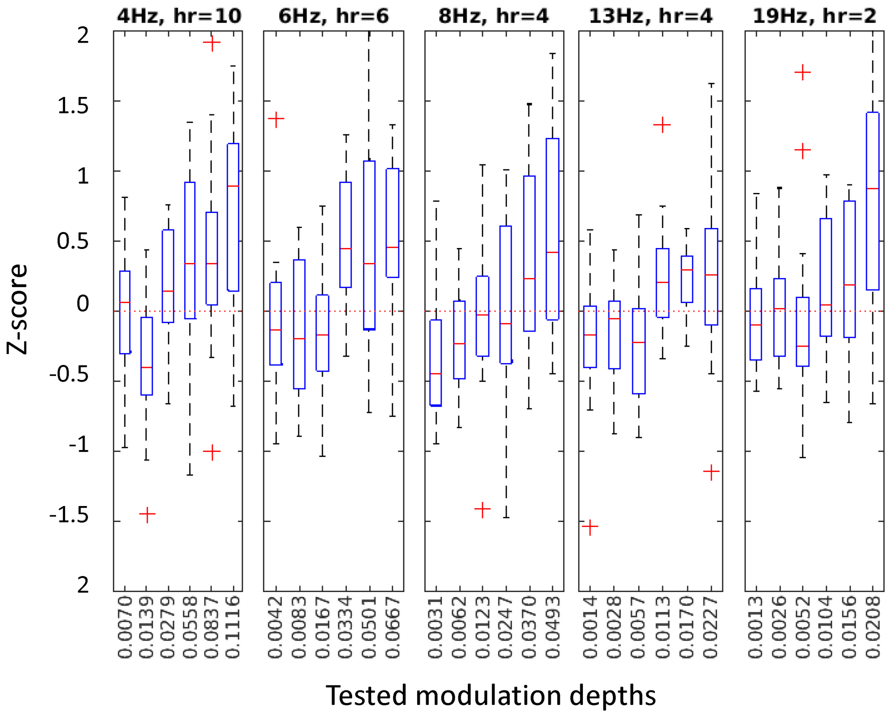

Figure S3. *z-score* distributions at the best harmonic component for each condition in Experiment 4. Different frequencies with their selected harmonic component in separate panels, tested MDs on the horizontal axes.
